# Controlled release spatial repellent devices (CRDs) as novel tools against malaria transmission: a semi-field study in Macha, Zambia

**DOI:** 10.1186/s12936-018-2558-0

**Published:** 2018-11-26

**Authors:** Jennifer C. Stevenson, Limonty Simubali, Twig Mudenda, Esther Cardol, Ulrich R. Bernier, Agustin Abad Vazquez, Philip E. Thuma, Douglas E. Norris, Melynda Perry, Daniel L. Kline, Lee W. Cohnstaedt, Pablo Gurman, Sebastian D’hers, Noel M. Elman

**Affiliations:** 1 Macha Research Trust, P.O. Box 630166, Choma, Zambia; 2 The W. Harry Feinstone Department of Molecular Microbiology and Immunology, The Johns Hopkins Malaria Research Institute, Johns Hopkins Bloomberg School of Public Health, 615 North Wolfe Street, Baltimore, MD 21205 USA; 3 Radboud University Nijmegen, Comeniuslaan 4, 6525 HP Nijmegen, Netherlands; 4 United States Department of Agriculture-Agricultural Research Service, Center for Medical, Agricultural, and Veterinary Entomology, 1600 SW 23rd Drive, Gainesville, FL 32608 USA; 5 Instituto Tecnológico de Buenos Aires (ITBA), Av. Madero 399, Ciudad Autónoma de Buenos Aires, Buenos Aires, Argentina; 6 Textile Materials Evaluation Team, The US Army Natick Soldier Research and Development Engineering Center (NSRDEC), Natick, MA USA; 7 United States Department of Agriculture-Agricultural Research Service, The Arthropod-Borne, Animal Diseases Research Unit (ABADRU), Manhattan, KS USA; 8 GearJump Technologies, LLC, P.O. Box 1600, Boston, MA 02446 USA

**Keywords:** Malaria, Mosquitoes, Spatial repellent, Semi-field-system, Zambia, Controlled delivery, Controlled release

## Abstract

**Background:**

The emergence of mosquitoes that can avoid indoor-deployed interventions, such as treated bed nets and indoor residual spraying, threatens the mainstay of malaria control in Zambia. Furthermore, the requirement for high coverage of these tools poses operational challenges. Spatial repellents are being assessed to supplement these vector control tools, but limitations exist in the residual effect of the repellent and the need for external power or heat for diffusion of the volatiles.

**Methods:**

A semi-field evaluation of a novel controlled release spatial repellent device (CRD) was conducted in Macha, Zambia. These devices emanate metofluthrin with no need for external power. Devices were deployed in huts within the semi-field system (SFS). Female *Anopheles gambiae* sensu stricto released within the SFS were trapped overnight by light traps and collected by aspiration the next morning inside and outside of huts to determine the extent of mosquito repellency and the impact on host-seeking and survival. Experiments studied the impact of number of devices as well as the presence of hut occupants. The study was complemented with numerical methods based on computational fluid dynamics to simulate spatial distribution of metofluthrin.

**Results:**

Presence of CRDs was associated with significant reductions in indoor counts of mosquitoes, regardless of whether huts were occupied or not. Repellency ranged from 15 to 60% compared to huts with no devices. Reducing the number of devices from 16 to 4 had little impact on repellency. When huts were occupied, indoor mosquito host-seeking was higher in the presence of CRDs, whilst survival was significantly reduced.

**Conclusions:**

This study demonstrated that deployment of as few as four CRDs within a hut was associated with reduced indoor mosquito densities. As would be expected, presence of occupants within huts, resulted in greater indoor catches (both with and without devices). The increased indoor mosquito host-seeking and mortality in huts when devices were present may be explained by the excito-repellency activity of metofluthrin. These semi-field experiments provide preliminary data on the utility of CRD spatial repellents to reduce indoor densities of *An. gambiae* mosquitoes. Studies will further investigate the impact of CRDs on mosquito behaviour as well as epidemiological protective efficacy.

**Electronic supplementary material:**

The online version of this article (10.1186/s12936-018-2558-0) contains supplementary material, which is available to authorized users.

## Background

Considerable gains have been made in the past 15 years in reducing malaria transmission globally, largely due to widely applied vector control measures including insecticide-treated bed nets (ITNs) and indoor residual spraying (IRS) [1–3]. Despite intensive scale-up of long-lasting insecticide-treated bed nets (LLINs) and annual IRS since the early 2000s, [4] malaria remains one of the primary causes of morbidity and mortality in children under 5 years in Zambia [5–7]. Unfortunately, these mainstays of vector control are threatened by mosquito resistance to insecticides and changes in mosquito behaviour that may result in increased outdoor foraging [8, 9]. Zambia has set a goal of eliminating malaria in the country by 2021, with the first-line areas targeted to become malaria-free being in the southern part of the country (Zambia National Malaria Elimination Centre, Lusaka *pers comm.*). Presently, however, the only vector control tools being deployed at scale are LLINs and IRS. The existence of mosquitoes that can feed around the times of bed net use, or do so outdoors, may be partly responsible for maintaining transmission in the southern part of the country [10]. Elimination of malaria will require additional new vector control approaches [1, 11, 12].

Spatial repellents (SRs) are typically based on pyrethroids, the same family of active ingredients (AIs) that are employed in IRS and for LLINs, but can be distinguished from insecticide formulations by the dosage or concentration used, the impact they have on targeted vectors, contact irritancy, and toxicity [13–15]. SRs interfere with the host-seeking process and biting of mosquitoes, and drive mosquitoes away from a treated space [16]. This elicited behaviour occurs at low vapour phase concentration. In contrast, insecticides that cause irritancy and kill mosquitoes, generally require higher doses. Unlike contact repellents that are applied to a surface and require mosquitoes to make physical contact, spatial repellents can reduce mosquito density and ultimately human-vector contact over a larger area as long as the AI concentration in air is high enough to repel or kill vectors. While the efficacy of tools such as LLINs is dependent on matching usage times with mosquito host-seeking and biting times, SRs have the potential to offer protection in areas with varied vector behaviour [17]. This particular feature is of special importance as several studies in sub-Saharan Africa have revealed vectors that forage outdoors and/or during early morning and evening [11, 18–22].

A number of studies have evaluated the entomological and epidemiological impact of SRs against various vector-borne diseases. Impacts have been seen on a range of mosquito behaviours both indoors and outdoors. In Belize, fewer mosquitoes were found to enter experimental huts when SRs were present [13, 23] and oviposition of *Aedes aegypti* was reduced following exposure to transfluthrin-impregnated strips in laboratory studies [24]. Human landing rates of anopheline mosquitoes were more than 90% lower when transfluthrin-treated hessian material was introduced in experimental flight tunnels [25], as well as in outdoor settings in urban Dar es Salaam and rural Ifakara, Tanzania [26, 27]. Early work in the same urban setting demonstrated reduced foraging with the use of transfluthrin-volatizing lamps set inside houses [28]. Laboratory and field studies in Indonesia, USA, Kenya, Vietnam and Cambodia similarly reported lower mosquito foraging indoors and outdoors with use of metofluthrin-impregnated materials and commercially available emanators [29–34]. The use of commercial emanators with metofluthrin in experimental rooms in houses in Australia resulted in almost complete inhibition of mosquito exposure due to increased knock-down, kill and disorientation of *Aedes* spp. [35]. These and other studies have demonstrated efficacy against mosquitoes from three major vector genera (*Anopheles* spp., *Culex* spp. and *Aedes* spp.), in various disease-transmission settings, as well as against mosquitoes that are active outdoors, bite in the early evening and that are insecticide resistant. Few studies have investigated the epidemiological impact of SRs. Burning of mosquito coils impregnated with such repellents has long been associated with reduction in mosquito bites, and their protective efficacy against malaria as well as mosquito bites have been demonstrated in randomized control trials in Indonesia, China and Bolivia [17, 36, 37]. Since spatial repellents employ lower concentrations of insecticides, they are expected to exert a lower selection pressure on emergence and/or spread of insecticide resistance alleles and phenotypes. They can also be deployed as an additional tool in combination with LLINs and IRS [36, 37].

The use and efficacy of commercially available SR devices, however, is often hampered by the need for an external power source (heat or electricity) and the short lifespan which necessitates frequent replacement. Mosquito coils are relatively inexpensive, but they represent a fire hazard, release toxic fumes presenting a health risk, and they are limited in duration to 4–12 h requiring regular replacement, which increases overall cost [14, 15, 38]. Some trials of repellent impregnated materials have been shown to have greater residual effects; transfluthrin treated hessian strips were shown to still impact mosquito host-seeking for up to 6 months post deployment in a semi-field setting, 3 months in Dar es Salaam, Tanzania [26] and up to a year in rural Tanzania [27]. Whilst these strips can be made relatively easily with little required technology, more practical easily deployable devices are still needed for protection in a diverse range of scenarios. There is an impending need for devices that are cost-effective, safe, battery-free, and long-lasting, that can be deployed easily within the community without significant training, and are effective indoors, in open-air structures and outdoors.

Recent advances in controlled release systems have allowed the implementation of new delivery of SRs integrating micro-systems, electronics and micro-electro-mechanical-systems (MEMS). These small form factor systems can be easily adapted as smart wearable devices for personal use, as well as implemented as field use devices with large payloads. This new generation vector-control system is designed to tune release kinetic profiles to optimize overall protection. MEMS-based devices can also be integrated with sensors for closed-loop operation to obtain an autonomous protection system [39, 40]. Controlled release devices (CRDs) can be designed to provide persistence for prolonged spatial protection. With funding from the Bill and Melinda Gates Foundation, GearJump Technologies have developed a controlled-release emanatory device containing metofluthrin, a pyrethroid which is in use in commercially available devices (Sumione^®^, Eminesce^®^, Sumitomo, JP) and registered for pesticide use in several countries [41]. CRDs release SRs over prolonged periods and can be easily deployed across indoor, semi-outdoor and outdoor settings, do not require batteries to operate, and do not present a fire hazard. Preliminary studies of CRDs in cage trials and semi-field systems conducted at the USDA in Gainesville, Florida, USA, have provided promising results against *Aedes aegypti* and *Anopheles quadrimaculatus.* To assess the efficacy of CRDs against African malaria vectors under natural field conditions, a semi-field study was conducted in Macha, Zambia. The main goal was to estimate the protective efficacy against indoor and outdoor host-seeking *Anopheles gambiae* sensu stricto (s.s.), one of the most important vector of malaria in sub-Saharan Africa.

## Methods

### Semi-field system and study site

**Fig. 1:**
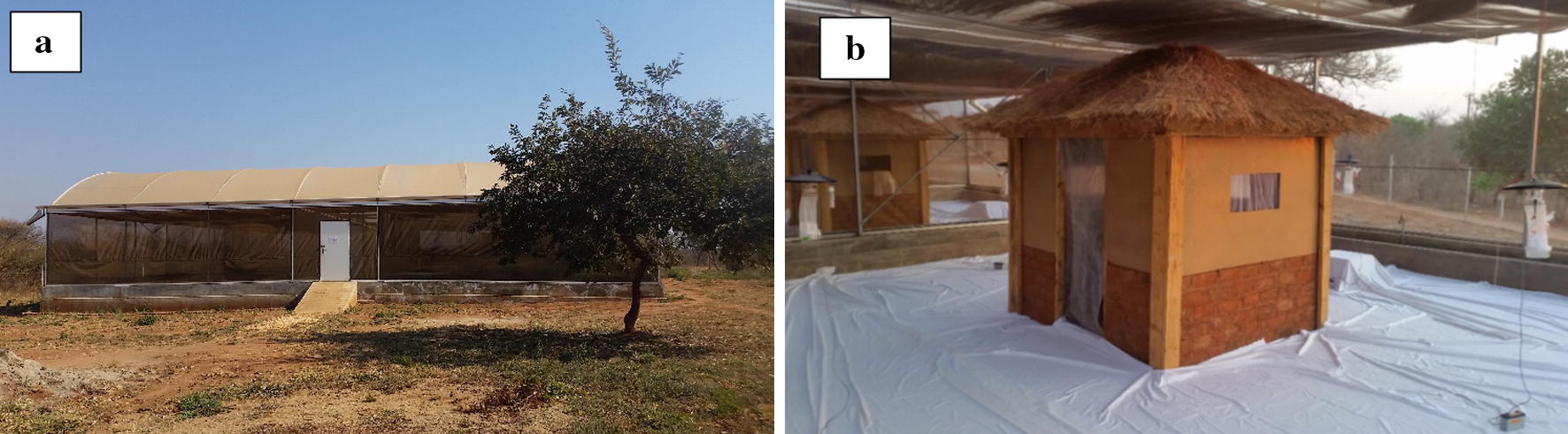
The semi-field system (SFS) at Macha, southern Zambia and the artificial huts constructed within the SFS. **a** Exterior view of the SFS. **b** Experimental set up depicting artificial huts and trap arrangement

Experiments were conducted in a semi-field system (SFS) at Macha Research Trust (MRT), Macha, southern Zambia. This SFS is a large, fully screened mosquito-proof greenhouse constructed on a concrete slab, (Fig. 1a) similar to those established in Tanzania for mosquito research [42–44]. The screen walls prevent escape of study mosquitoes and entry of wild mosquitoes, other insects and animals, whilst allowing for normalization of natural climatic conditions with that of the external environment. The SFS measures 28.8 m × 21 m with three chambers of 9 m × 9.5 m on each side separated by a central corridor. For this study, chambers with a concrete base fitted with ‘moats’ to prevent ant entry on one side of the SFS were used. Huts measuring 2 m (l) × 2 m (w) × 3 m (h), with open eaves, resembling house structures present in the rural community of Macha were constructed in each chamber. Door openings were covered with a sheet of plastic while non-impregnated netting were hung in front of the windows to serve as curtains. The floor of each chamber was covered with white sheeting to easily observe knocked down mosquitoes (Fig. 1b).

### Controlled release device

**Fig. 2:**
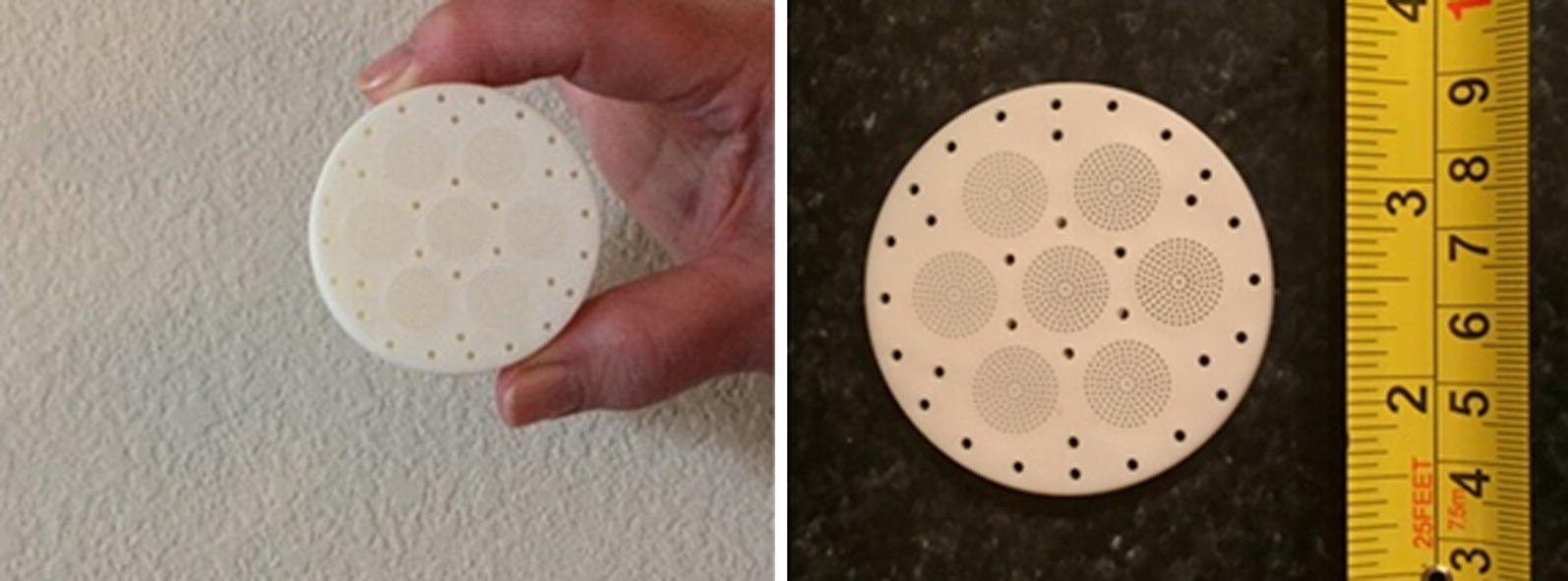
The controlled release spatial repellent device (CRDs). This plastic encased device measure 5.5 cm in diameter and 2.5 cm in height and contained ~ 3.5 mL of AI

The SR CRD was manufactured by GearJump Technologies, LLC. and contained ~ 3.5 mL of the active ingredient metofluthrin at 30% v/v dissolved in isopropyl alcohol 70% v/v. The CRD exterior measuring 5.5 cm in diameter and 2.5 cm in height was made of polymeric material for this study, but could also potentially be made of biodegradable polymers. No external power source was needed to release the SR from the CRD (Fig. 2); an internal exothermic reaction increased volatilization of the SR following an initial activation, by internally increasing the local temperature of the AI chamber by 7–10 °C over a period of 16–24 h.

### Mosquitoes

*Plasmodium*-free, insecticide-susceptible female *An. gambiae* s.s. mosquitoes (Kisumu strain) aged 2–5 days old were used in this study. These mosquitoes were reared in the MRT insectary at approximately 28 °C, 80% RH and under a 12:12 h light/dark cycle using standard mosquito rearing protocols. Mosquitoes were starved of glucose for a period of 6 h before commencement of the experiments to encourage host-seeking.

### Hut occupants

For the final set of experiments that involved collection of mosquitoes from occupied huts, trained staff slept under an untreated bed net for the night. All occupants were African men between 30 and 40 years of age who verbally consented to participate and signed agreements stating their roles. Each was screened for malaria prior to and every 2 weeks during the study. They were informed that they may experience discomfort from mosquito bites if the net was not used properly, but that the mosquitoes used were uninfected colony insects. Each occupant was assigned a hut to occupy every night of the experiment and did not move from hut to hut such that variability in attractiveness and variation between chambers was grouped and accounted for in analyses.

### Experimental design and set-up

**Fig. 3:**
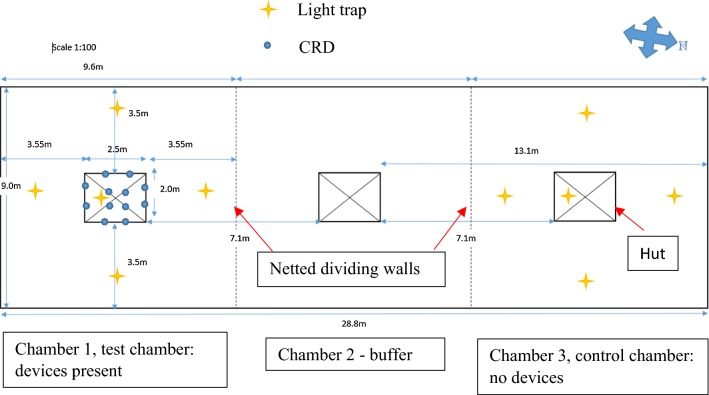
Experimental set up. Floor plan of lay out of huts, devices and traps within the SFS at Macha. Example shows set up of Experiment 1 with 12 devices in the eaves and 4 suspended from the rafters within the hut

**Fig. 4:**
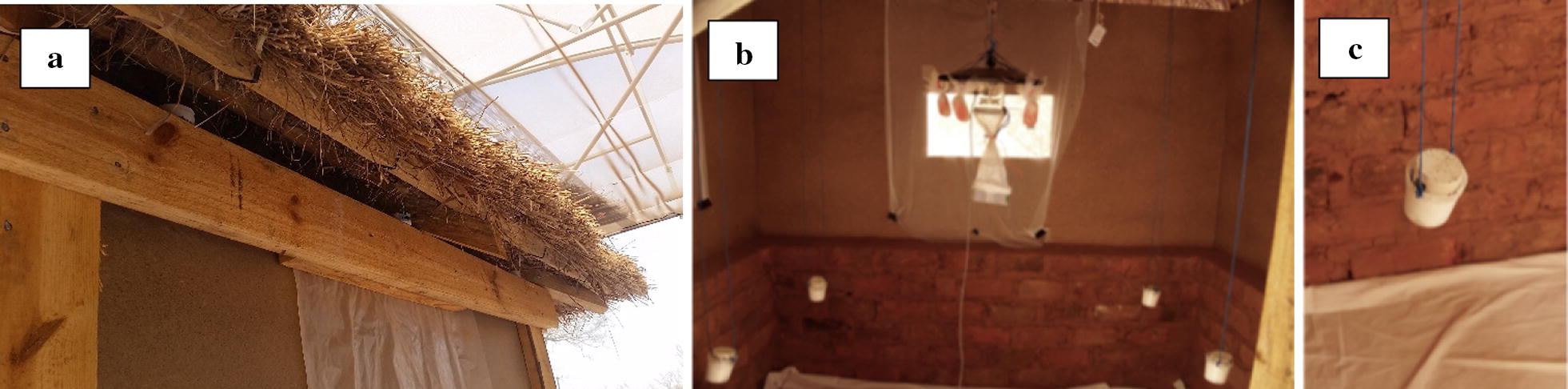
Placement of CRDs in **a** eaves and **b**, **c** suspended from the rafters of the hut within the semi-field system

Two outer chambers (1 and 3) of three neighbouring compartments of the SFS were used for the experiments. One of the outer chambers was used for the active group where the CRDs were placed, while the other outer chamber farthest away was used for the control group without CRDs. The middle chamber served as buffer to prevent cross contamination of the emitted repellent from the active group to the control group (Fig. 3). CRDs were rotated in a cross-over design between chamber 1 and 3 and each rotation replicated five times, such that each chamber received the devices five times in each of the three experiments. Initial assignment of the CRDs to a chamber was randomised for each rotation. Experiments were conducted twice per week with 2–3 days between experimental nights to allow for any residual repellent to dissipate. Experiments began in October 2016 and were completed in February 2017. During the study, climatic conditions within the SFS were logged using a HOBO^®^ weather station (Onset^®^ Computer Corporation, Bourne, MA USA) that recorded humidity and temperature every 15 min. From these data, the mean, minimum and maximum temperatures and humidity were calculated for each experimental night. Moon illumination for Zambia for each study night was acquired from the Astronomical Applications Department of the US. Naval Observatory. Staff documented wind levels as still, light, medium or strong at the start of each experiment. Chambers were prepared during the day and devices placed in the eaves of the huts and/or suspended from the ceiling (Fig. 4a–c) 6 h before the release of mosquitoes to allow the exothermic reaction within the CRDs to initiate and for metofluthrin to diffuse into the chamber space. Within each chamber, CDC light traps (John W. Hock Ltd., Gainesville, Florida, USA) with artificial bait (BG Lure^®^, Biogent AG, Regensburg, Germany) set at 1.5 m above the ground, were placed outside the hut 2 m from the exterior wall on all sides (4 per chamber) as a proxy for outdoor host-seeking rates. Indoors, one CDC light trap was suspended from the roof next to an untreated mosquito net hung over a mattress to measure host-seeking.

Three experiments were conducted as described below, including the amounts of the active ingredient dispensed in each hut following the set up:

Experiment 1, huts unoccupied: Active chamber: 4 CRDs were suspended from the rafters of the hut set 0.7 m from the ground and 12 CRDs were placed in the eaves of the same hut. Eaves were selected as they are considered important entry points for mosquitoes. Total metofluthrin mass per unit chamber volume released: 5.65 g/m^3^. Control chamber: no devices were used.

Experiment 2, huts unoccupied: Active chamber: 4 CRDs were suspended from the rafters of the hut at 0.7 m from the ground. Total metofluthrin mass per unit chamber volume released: 1.41 g/m^3^. Control chamber: no devices were used.

Experiment 3, huts occupied from 18:00 to 06:00 during experimental nights with a staff member sleeping in each hut on a mattress under an untreated mosquito net hung in the centre of the hut: Active chamber: 4 CRDs were suspended from the rafters of the hut at 0.7 m from the ground. Total metofluthrin mass per unit chamber volume released: 1.41 g/m^3^. Control chamber: no devices were used.

### Mosquito release and collections

Each night of the experiment equal numbers of mosquitoes were released into each chamber at 17:45, with a minimum of 100 and maximum of 300 being released into each chamber on a single night. Light traps were switched on at 18:00 and switched off at 06:00 h the following morning. Traps were retrieved and dead and alive mosquitoes were collected from inside to outside the huts using aspirators. Mosquitoes found alive, both in traps and from morning aspiration collections, were killed by freezing. All captured mosquitoes in each chamber were counted, sorted by location (indoor/outdoor traps, inside/outside huts), and marked as dead or alive.

### Data analysis

The entomological endpoints reported for this study were as follows:

- *Live in hut* Number of live mosquitoes caught inside the hut the morning after the experiment (excluding those captured inside the indoor CDC light trap).
- *Live outside hut* Number of live mosquitoes caught outside the hut the morning after the experiment (excluding those captured in the outdoor CDC light traps).
- *Dead in hut* Number of dead mosquitoes found inside the hut the morning after the experiment (excluding those captured inside the indoor CDC light trap).
- *Dead outside hut* Number of dead mosquitoes found outside the hut the morning after the experiment (excluding those captured in the outdoor CDC light traps).
- *Host*-*seeking indoors* Number of mosquitoes caught in the indoor trap which ran from 18:00 to 06:00.
- *Host*-*seeking outdoors* Total number of mosquitoes caught in the four outdoor traps which ran from 18:00 to 06:00.
- *Total indoors* Total number of mosquitoes collected in the hut i.e. ‘Live in hut’ + ‘Dead in hut’ + ‘Host-seeking indoors’.
- *Total outdoors* Total number of mosquitoes i.e. ‘Live outside hut’’ + ‘Dead outside hut’ + ‘Host-seeking outdoors’.

Graphical representations of the data are shown as proportion of captured mosquitoes by location.

The number of mosquitoes caught in various positions or in traps with or without the SR device in place was compared using generalized linear models (GLMs) using a Poisson distribution with logit link function. The dependent variables investigated were the number of mosquitoes caught host-seeking indoors or outdoors (i.e. those captured in the traps), the total indoor or total outdoor catch, and the total number found dead both indoors and outdoors taking into account the number of mosquitoes captured, with the independent variables being the treatment (presence or absence of CRD), climatic conditions (mean, minimum and maximum nightly temperatures and humidity), moon illumination, wind speed (as documented subjectively by study staff), chamber used and day of the experiment. All analyses were carried out in STATA^®^ (v13.1, Stata Corp., Texas, USA).

In addition, the reduction in host-seeking, both indoors and outdoors, and reduction of indoor or outdoor catches was assessed by adapting the WHO calculation [45] to estimate percentage inhibition as follows:

$$\varvec{\% inhibition} = \left[ {\frac{{(\varvec{C} - \varvec{T})}}{{\left( \varvec{C} \right)}}} \right] \times 100$$

where *C* is the number of mosquitoes host-seeking or total indoor/outdoor catch in the control chamber and *T* is the number of mosquitoes in the treatment space.

### Simulations

**Fig. 5:**
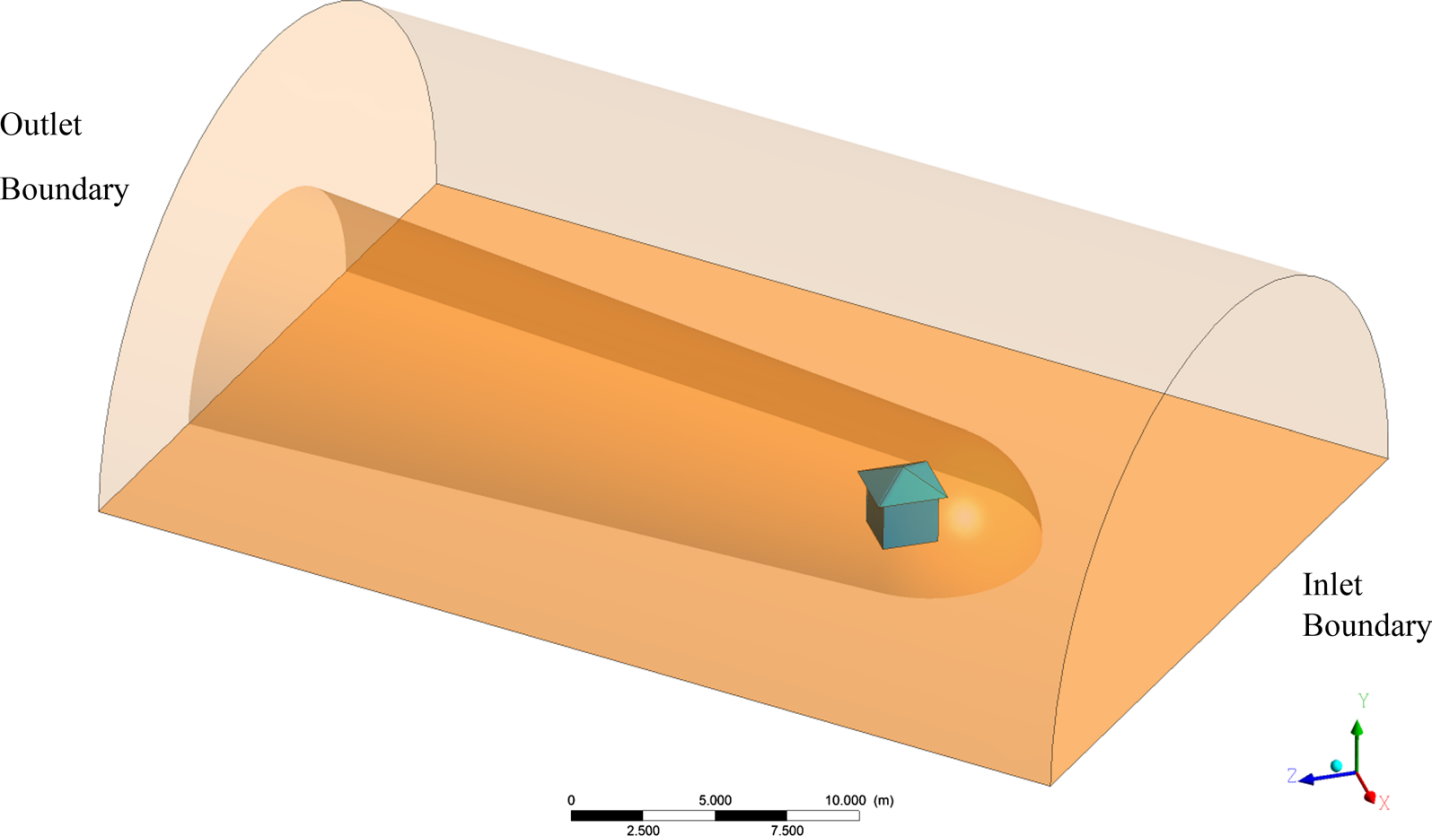
Computational fluid dynamics model simulation domain

A computational fluid dynamics (CFD) model was developed to estimate metofluthrin concentration in the hut and its surroundings. A 3D domain with the hut geometry integrated inside was considered. The inlet boundary and external tangent to cylinder surface velocity as well as outlet boundary pressure were fixed to assess wind effect (Fig. 5). The hut was placed inside of the 3D domain with the correct angle to consider wind direction. Two mesh refinements were implemented, one finer near the hut field and the other one coarser in the far field. The turbulence adopted model was k-epsilon. The domain extension was enlarged to allow wind to reach fully developed state. A transport model was used to track metofluthrin concentration in the domain considering diffusion and convection. Kinematic diffusivity of metofluthrin was set to 6.8e−06 m^2^/s. CRDs were modelled as point sources with a fixed metofluthrin mass release rate, which was set to 0.224 mg/s per device as determined from previous in vitro evaporation tests based on gravimetric analysis. The resulting concentration distribution of metofluthrin in air was simulated and evaluated to find a protective volume where a threshold concentration was exceeded. The boundary protective surface was defined as where the concentration meets the threshold value, set to 0.234 ppm as obtained for *An. quadrimaculatus* in a prior study that correlated with mosquito mortality and spatial concentration distribution of metofluthrin in a 24 h and 48 h semi-field study (Elman et al. pers.comm.). This approach provides a powerful tool to define the target concentration of metofluthrin based CRDs release rate, potentially allowing optimization of deployment ahead of field studies.

## Results

### Semi-field experiments

#### Experiment 1: 16 devices, 12 on the eaves, 4 hanging inside from the rafters, hut unoccupied

**Fig. 6:**
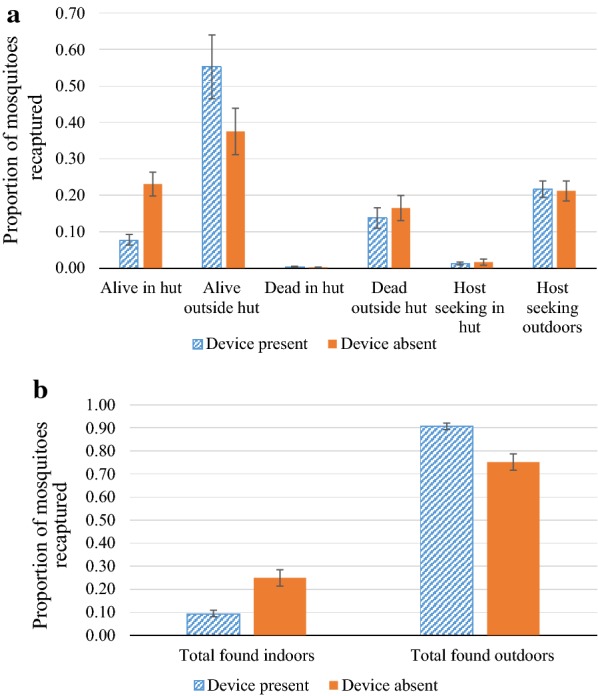
Results of Experiment 1: 12 CRDs in the eaves and 4 suspended from the rafters of the hut. **a** Comparison of proportion of *An. gambiae* s.s. mosquitoes captured in different locations within the SFS with and without devices. **b** Comparison of proportion of *An. gambiae* s.s. mosquitoes captured indoors and outdoors with and without devices

The number and proportion of captured mosquitoes caught at the various localities are displayed in Table 1 and Fig. 6a. When calculating the percentage difference in proportions caught when the devices were in place across all rotations, the presence of the CRDs resulted in a 24% reduction in indoor host-seeking (as determined by the baited indoor light trap catches), and a 62% reduction in the proportion of total mosquitoes found indoors. Conversely, outdoor host-seeking increased by 2% and total mosquitoes outside of the hut increased by 20% (Fig. 6b). After controlling for temperature, humidity, moonlight intensity, wind, selected chamber and the date of the experiment, the presence of devices was significantly associated with a reduction in indoor total catch [Odds Ratio (OR) = 0.32, 95% CI = 0.25, 0.40, p < 0.001], but the association with reduction in indoor host-seeking was not significant (OR = 0.58, 95% CI = 0.28, 1.20, p = 0.144). Outdoors, the increase in host-seeking and outdoor catch was not statistically significant (outdoor host-seeking: OR = 1.04, 95% CI = 0.86, 1.26, p = 0.675; outdoor total catch: OR = 0.96, 95% CI = 0.80, 1.15 p = 0.655). Interestingly, with devices present, the total number of dead mosquitoes both indoors and outdoors was significantly reduced (OR = 0.78, 95% CI = 0.63, 0.98 p = 0.035).

**Table 1:** Experiment 1: Impact of CRDs on indoor and outdoor catch, foraging and mortality of mosquitoes

	Outcomes	Number of mosquitoes (% of those captured)		Odds ratio	95% CI	p
		CRD present (captured n = 1378)	CRD absent (captured n = 1311)			
Indoor	Total catch^a^	126 (9.1)	322 (24.6)	0.32	0.25, 0.40	*0.001*
	Foraging^b^	16 (1.2)	20 (1.5)	0.58	0.28, 1.20	0.144
Outdoor	Total catch^a^	1252 (90.9)	989 (75.4)	0.96	0.80, 1.15	0.655
	Foraging^b^	295 (21.4)	260 (19.8)	1.04	0.86, 1.26	0.675
Indoors and Outdoors	Dead	188 (13.6)	205 (15.6)	0.78	0.63, 0.98	*0.035*

In Experiment 1, Twelve CRDs were placed in the eaves and 4 were hung from the rafters of the house in the SFS. There were no occupants in the hut. CRDs were alternated in a cross-over design and impacts on released female *An. gambiae* s.s. mosquitoes studied over 10 experimental nights. Number captured refers to total numbers retrieved the following morning from all study nights. Odds ratios were generated from generalized linear models (GLMs) using a Poisson distribution with logit link function comparing of number of mosquitoes collected with or without the CRD

Italic values indicate significance of p value (p < 0.05)

^a^Total catch represents sum of foraging mosquitoes caught in light traps, and those found alive or dead the next morning

^b^Foraging represents total caught in light traps

#### Experiment 2: 4 devices hanging inside from the rafters, hut unoccupied

**Fig. 7:**
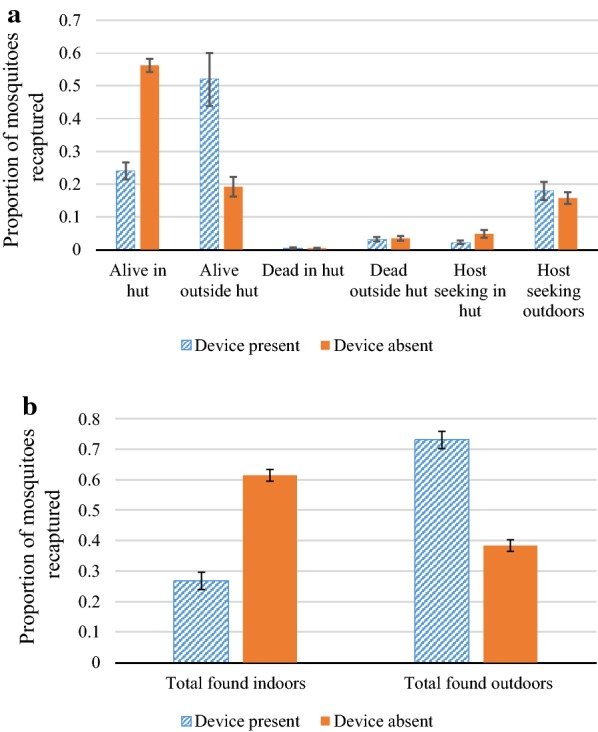
Results of Experiment 2: 4 CRDs suspended from the rafters of the hut. **a** Comparison of proportion of *An. gambiae* s.s. mosquitoes captured in different locations within the SFS with and without devices. **b** Comparison of proportion of *An. gambiae* s.s. mosquitoes captured indoors and outdoors with and without devices

When only four devices were deployed, the reduction in indoor host-seeking and indoor catch compared to controls was less compared to results from Experiment 1, but still sizeable with a 54% reduction in host-seeking indoors and a 56% reduction in total indoor catch (Table 2, Fig. 7a). In the multivariable model controlling for environmental conditions and chamber and day, the presence of the devices was significantly associated with a reduction in both indoor host-seeking and the total indoor catch (indoor host-seeking: OR = 0.34, 95% CI = 0.22, 0.53 p < 0.001; total indoors: OR = 0.20, 95% CI = 0.17, 0.24 p < 0.001). Outdoors, host-seeking increased by 14% and the total captured outside the hut increased by 90% when the CRDs were present compared to when the devices were absent (Fig. 7b), however these increases were not statistically significant once other variables had been accounted for (outdoor host-seeking: OR = 1.06, 95% CI = 0.87, 1.30 p = 0.560; outdoor total catch: OR = 1.10, 95% CI = 0.91, 1.34, p = 0.332). There was no statistically significant association between the presence of devices and total number of mosquitoes killed (OR = 1.05, 95% CI = 0.711, 1.56, p = 0.800).

**Table 2:** Experiment 2: Impact of CRDs on indoor and outdoor catch, foraging and mortality of mosquitoes

	Outcomes	Number of mosquitoes (% of those captured)		Odds ratio	95% CI	p
		CRD present (captured n = 1410)	CRD absent (captured n = 1437)			
Indoor	Total catch^a^	361 (25.6)	900 (62.6)	0.20	0.17, 0.24	*0.001*
	Foraging^b^	28 (2.0)	81 (5.6)	0.34	0.22, 0.53	*0.001*
Outdoor	Total catch^a^	1049 (74.4)	537 (37.3)	1.10	0.91, 1.34	0.332
	Foraging^b^	234 (16.6)	228 (15.9)	1.06	0.87, 1.30	0.560
Indoors and Outdoors	Dead	55 (3.9)	52 (3.6)	1.05	0.71, 1.56	0.800

In Experiment 2, Four CRDs were hung from the rafters of the house in the SFS. There were no occupants in the hut. CRDs were alternated in a cross-over design and impacts on released female *An. gambiae* s.s. mosquitoes studied over 10 experimental nights. Number captured refers to total numbers retrieved the following morning from all study nights. Odds ratios were generated from generalized linear models (GLMs) using a Poisson distribution with logit link function comparing of number of mosquitoes collected with or without the CRD

Italic values indicate significance of p value (p < 0.05)

^a^Total catch represents sum of foraging mosquitoes caught in light traps, and those found alive or dead the next morning

^b^Foraging represents total caught in light traps

#### Experiment 3: 4 devices hanging inside from the rafters, hut occupied

**Fig. 8:**
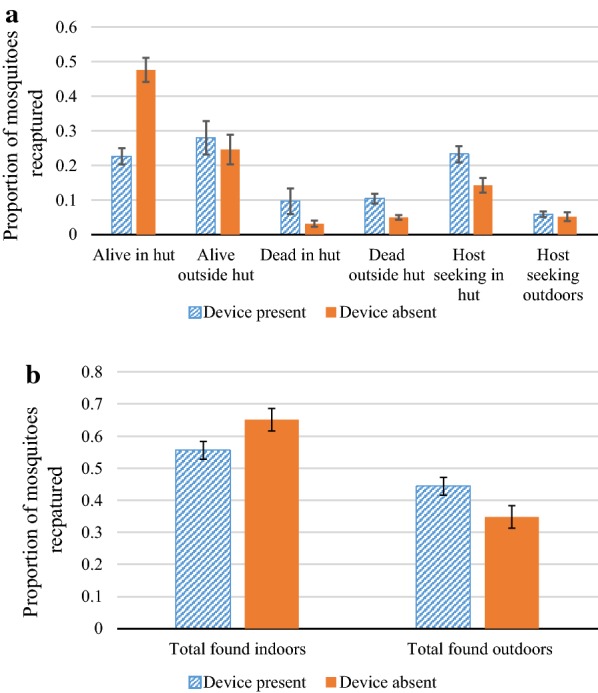
Results of Experiment 3: 4 CRDs suspended from the rafters of the hut, huts occupied. **a** Comparison of proportion of *An. gambiae* s.s. mosquitoes captured in different locations within the SFS with and without devices. **b** Comparison of proportion of *An. gambiae* s.s. mosquitoes captured indoors and outdoors with and without devices

When the huts were occupied the overall indoor catch, regardless of presence or absence of the devices, increased greatly as indoor host-seeking increased, as would be expected due to the presence of a human as an attractant. In the multivariable GLM, having controlled for presence of devices and environmental or chamber/day effects, the presence of people in this third experiment was associated with an almost five-fold increase in indoor host-seeking compared to Experiment 2 (OR = 4.88, 95% CI: 3.60, 6.61, p < 0.001) (Additional file 1). The presence of devices was associated with a 63% increase in indoor host-seeking compared to when devices were absent, however overall indoor catch which included those host-seeking indoors and those resting inside or found dead indoors the next morning, was reduced overall by 15% (Table 3, Fig. 8a, b). These associations were both statistically significant when other factors were accounted for in the model (indoor host-seeking: OR = 1.87, 95% CI 1.54, 2.25, p < 0.001; indoor catch OR = 0.66, 95% CI 0.57, 0.77, p < 0.001). Whilst presence of the devices was associated with an increase in outdoor host-seeking and total catch outdoors of 14% and 27%, respectively, neither of these were found to be statistically significant in the multivariable models (outdoor host-seeking: OR = 1.06, 95% CI = 0.78, 1.45, p = 0.711; outdoor total catch: OR = 0.88, 95% CI = 0.66, 115, p = 0.350). Presence of devices, however, was significantly associated with an increased total number found dead (OR = 3.06, 95% CI = 2.43, 3.86, p < 0.001), with more than double the mosquitoes being found dead indoors the next morning than when CRDs were absent.

**Table 3:** Experiment 3: Impact of CRDs on indoor and outdoor catch, foraging and mortality of mosquitoes

	Outcomes	Number of mosquitoes (% of those captured)		Odds ratio	95% CI	p
		CRD present (captured n = 1508)	CRD absent (captured n = 1477)			
Indoor	Total catch^a^	842 (55.8)	971 (65.7)	0.66	0.57, 0.77	*0.001*
	Foraging^b^	363 (24.1)	212 (14.3)	1.87	1.54, 2.25	*0.001*
Outdoor	Total catch^a^	666 (44.2)	506 (34.3)	0.88	0.66, 1.15	0.350
	Foraging^b^	89 (5.9)	82 (5.6)	1.06	0.78, 1.45	0.711
Indoors and Outdoors	Dead	306 (20.3)	119 (8.1)	3.06	2.43, 3.86	*0.001*

In Experiment 3, Four CRDs were hung from the rafters of the house in the SFS. Each hut was occupied by a person under an untreated bed net. CRDs were alternated in a cross-over design and impacts on released female *An. gambiae* s.s. mosquitoes studied over 10 experimental nights. Number captured refers to total numbers retrieved the following morning from all study nights. Odds ratios were generated from generalized linear models (GLMs) using a Poisson distribution with logit link function comparing of number of mosquitoes collected with or without the CRD

Italic values indicate significance of p value (p < 0.05)

^a^Total catch represents sum of foraging mosquitoes caught in light traps, and those found alive or dead the next morning

^b^Foraging represents total caught in light traps

#### Overall impact of presence of CRDs

Having controlled for all environmental variables, the timing of each experiment and the type of experiment in the model, deployment of the devices was associated with an overall significant reduction in indoor catch, with the odds of entering huts being reduced 64% compared to having no devices present (OR = 0.36 95% CI = 0.33, 0.40; p < 0.001), regardless of the number of devices used (Table 4).

**Table 4:** Global analysis of Experiments 1–3: Impact of CRDs on indoor and outdoor catch, foraging and mortality of mosquitoes

	Outcomes	Number of mosquitoes (% of those captured)		Odds ratio	95% CI	p
		CRD present (captured n = 4296)	CRD absent (captured n = 4225)			
Indoor	Total catch^a^	1329	2193	0.36	0.33, 0.40	*0.001*
	Foraging^b^	407	313	1.31	1.12, 1.55	*0.001*
Outdoor	Total catch^a^	2967	2009	1.01	0.89, 1.13	0.912
	Foraging^b^	618	570	1.05	0.93, 1.20	0.364
Indoors and Outdoors	Dead	549	376	1.47	1.27, 1.69	*0.001*

Odds ratios were generated from generalized linear models (GLMs) using a Poisson distribution with logit link function comparing of number of mosquitoes collected with or without the CRD, accounting for experiment type, environmental variables and the timing of each experiment. For all experiments, CRDs were alternated in a cross-over design and impacts on released female *An. gambiae* s.s. mosquitoes studied over 10 experimental nights. Number captured refers to total numbers retrieved the following morning from all study nights

Italic values indicate significance of p value (p < 0.05)

^a^Total catch represents sum of foraging mosquitoes caught in light traps, and those found alive or dead the next morning

^b^Foraging represents total caught in light traps

### Simulations

The first semi-field experiment was simulated using the CFD model for which the devices were active for a period of 18 h. The simulations provided concentration distributions within a volume domain. Within this domain, an isosurface was then interpolated for metofluthrin concentration levels at 0.234 ppm (defined as the threshold concentration) in order to obtain a protective envelope.

**Fig. 9:**
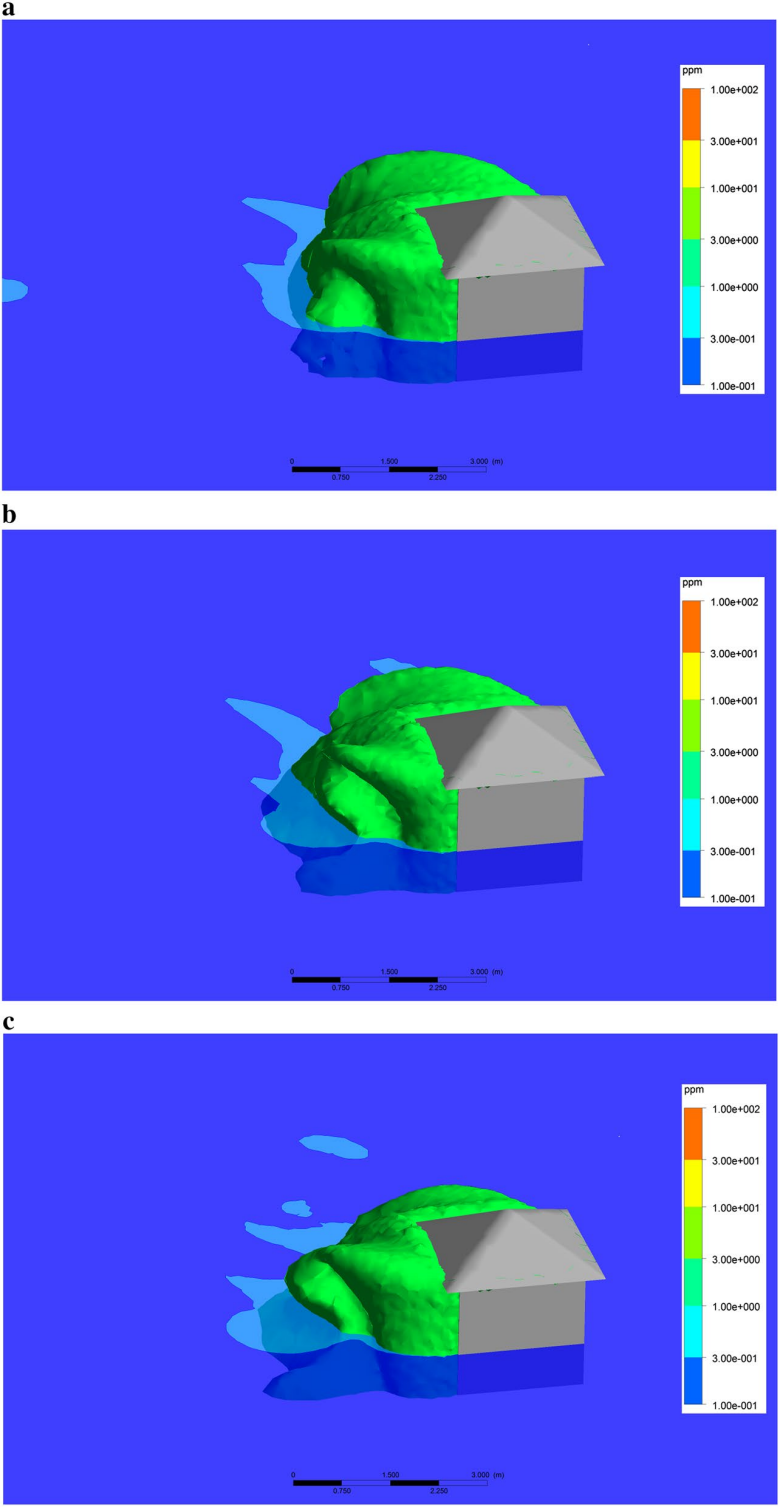
Simulations of protection plumes around huts using nominal release rates of metofluthrin. A Protection volume was defined using input parameters (metofluthrin release rates, environmental conditions) and simulated **a** prior to test initiation (1 h after devices are activated), **b** at test initiation (6 h after devices are activated), **c** at test end (18 h after devices are activated)

Additionally, concentration plots at a plane located 0.35 m above the floor were obtained to evaluate distribution of metofluthrin. Figure 9a, b and c shows the protective envelope and the concentration plots for the following times after device activation for the experiments: 1 h prior to initiation, 6 h after initiation, and 18 h post initiation at the study end.

**Fig. 10:**
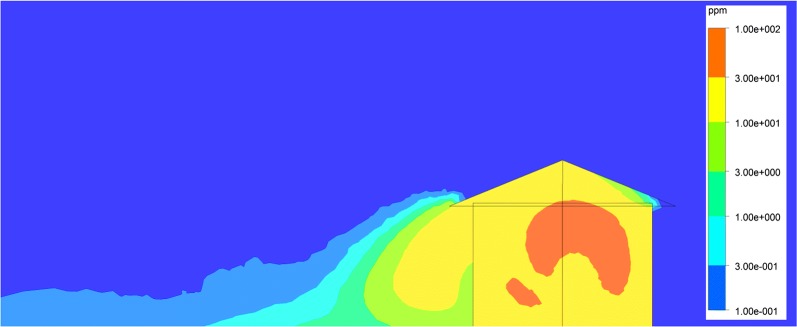
Metofluthrin concentrations within and outside the hut after 12 h (mass rate = 1 × 10^−8^ kg/s)

It was observed that the steady state is reached relatively quickly, suggesting that an hour is long enough to stabilize the metofluthrin concentration. Figure 10 shows concentrations within the hut and demonstrates that the threshold concentration of metofluthrin is reached within the hut. Partial protection is predicted outside up to the height of the hut due to emanation of the repellent from the eaves with a non-uniform area span, clearly controlled by wind direction.

## Discussion

This study demonstrated that the presence of SR CRDs suspended from the rafters of a hut was associated with a significant reduction in overall mosquito densities indoors, and a reduction of total indoor catch was evident with and without human occupants. The three experiments explored the impact of CRDs on mosquito densities, host-seeking and death with (a) numerous devices deployed both in the eaves and hanging indoors, with (b) only four devices hanging indoors and (c) having people occupy the hut through the night with four devices hanging indoors. Total indoor catch the following morning was significantly reduced in all three experiments when CRDs were deployed, suggesting that presence of devices leads to reduced mosquito exposure indoors. It was envisioned that the placement of twelve devices in the eave gaps and four devices placed indoors would result in a far greater reduction in indoor mosquitoes compared to just four hanging indoors. However, the reduction in impact (62% compared to 54% reduction in indoor catches), demonstrated that deployment of four devices would still result in a sizeable reduction in indoor exposure while reducing the expense. Simulation results show that the concentration threshold value was reached inside the hut for both scenarios. One possible explanation for the greater reduction in indoor catches in Experiment 1 is that the presence of CRDs in the eaves created a perimeter barrier reducing the number of mosquitoes that entered the hut.

The impact on host-seeking (as measured by light traps) when CRDs were present compared to controls, varied between the experiments. Reductions in indoor host-seeking were evident in the first two experiments when devices were deployed, although having controlled for other climatic and time variables, this reduction was only significant in Experiment 2. In Experiment 3, indoor host-seeking was significantly increased in the presence of indoor CRDs. One possible explanation for this increase is that the attractive presence of people in the hut led to higher mosquito densities indoors, increased flight activity of the mosquitoes resulted due to the excito-repellent effects of the pyrethroid used in CRDs, and with limited space for mosquitoes to disperse and leave resulted in higher light trap captures and greater mosquito mortality of mosquitoes. Overall indoor collections (i.e. those caught in light traps combined with those found dead or alive the next day) were significantly reduced when devices were in place due primarily to far fewer mosquitoes being captured alive. These findings corroborate those of previous laboratory and semi-field studies in Tanzania where transfluthrin coils increased activation of mosquitoes and, in combination with human volatiles, resulted in greater taxis to the host. Further experiments demonstrated that despite this apparent increased attraction, fewer mosquitoes successfully landed on people and feeding inhibition lasted several hours [46]. Further studies are required to determine whether similar responses would be seen with these metofluthrin devices and whether actual landing and probing, as opposed to host-seeking, would be inhibited.

In these experiments, outdoor traps were set two metres from huts to CRDs were deployed indoors at 0.7 m above the ground, below the level of the windows to optimize indoor repellent concentration levels. Simulations revealed that metofluthrin released by the CRDs placed in the eaves is dispersed quickly by air flow. In all experiments the proportion of mosquitoes host-seeking outdoors was not significantly different between control and active (presence of CRDs) chambers, suggesting that the repellent did not emanate at concentrations high enough to impact mosquitoes at distances of 2 m away from the hut. Studies using semi-field tunnels in Tanzania estimated the protective distance of burning mosquito coils and demonstrated that reduction in host-seeking is highest when devices are in close proximity to potential hosts but that substantial reductions in mosquito host-seeking are still evident up to 30 m from a point source [46]. The lack of impact on host-seeking outdoors close to where the CRDs were deployed in the current study could be because the airborne concentration of metofluthrin was too low to cause an effect. This warrants further investigation. As shown in other studies ventilation and wind direction and speed can greatly affect the impact of repellents [32, 38, 46, 47]. Although wind was controlled for in the analyses, documentation of this was subjective and future studies should employ anemometers [46]. Data on wind direction and speed could be integrated into future simulation models to better guide the numer and placment of emanators.

In Experiment 1 it was also shown that presence of 16 CRDs was associated with a significant reduction in mosquito mortality the following morning. Of the few found dead, almost all were found outdoors (only six mosquitoes were found dead indoors in both active and control chambers from 2689 captured over ten experimental nights). This study was conducted within a closed semi-field system, where the number of mosquitoes per capture location was bound by the total number released. One explanation for the reduction in mortality is that the presence of a large number of CRDs in the huts resulted in a high concentration of SR that prevented mosquitoes from entering, to these repelled mosquitoes were either trapped in outdoor traps or survived outdoors. Conversely, when huts were occupied in Experiment 3, the proportion found dead the next morning was greater both indoors and outdoors. As alluded to earlier, this is likely due to the combined presence of CRDs and human volatiles indoors. Induced mortality from SRs may occur when concentrations of the active ingredient build up in confined spaces or with limited ventilation [38], which may have been the case with the small huts used in the study. Direct measurements of repellent concentrations within the huts and indoor airflow would better support this hypothesis. The increased activation of mosquitoes in the presence of both human volatiles and the active compound [46] combined with the inability to feed on a host to replenish energy reserves or access water for necessary hydration, likely contributed to the higher mosquito mortality seen in the presence of the devices. Future studies should evaluate impacts on mosquitoes of deploying CRDs in larger structures with presence and absence of energy sources.

Occupation of huts resulted in more than a two-fold increase in indoor light trap catches compared to light traps alone with artificial baits, and a relative increase in host-seeking of almost five-fold. BG lures are primarily designed for *Aedes* mosquitoes, rather than anophelines that possess different odorant receptors and may demonstrate different chemosensory behaviours and attraction to volatiles [48, 49]. At the time of the study BG lures were the only manufactured artificial baits easily available for integration with light traps, which showed a relatively poor attraction of mosquitoes compared to a live human in this study. As such, it is likely that the outdoor traps, which were fitted only with the BG lure across all experiments, did not optimally trap outdoor host-seeking mosquitoes despite being standardized across all experiments. Future studies should focus on using human landing catches or more effective anopheline bait formulations, both indoors and outdoors, to better sample foraging mosquitoes and determine the extent to which the repellent interrupts this behaviour.

In all experiments, indoor density was defined as the total number of mosquitoes caught indoors in light traps fitted with artificial lures, combined with total number still found resting indoors the next morning or dead within the huts. The huts were not fitted with entry or exit traps that would have provided more detailed data on the impact of the CRDs on specific mosquito activity such as reduced house entry or greater house exit, indicative of repellency. Future experiments should determine the impact on a larger range of mosquito behaviours.

In southern Zambia, the primary vector of human malaria is *Anopheles arabiensis* [50], a vector known to exhibit markedly different foraging behaviours than that of *An. gambiae* s.s. This species is generally reported to be more plastic in its behaviours, feeding on both animals and humans, indoors and outdoors [39, 40]. Macha Research Trust is establishing a colony of this species. There would be value in assessing the impact of the CRDs against this local vector and additional emerging vectors suspected of foraging primarily outdoors. Furthermore, *An. gambiae* s.s. Kisumu is an insecticide-susceptible strain of mosquito that has been in colony for almost four decades and as such is highly inbred and likely to demonstrate different and perhaps more consistent behaviours as compared to wild populations which undoubtedly maintain a higher degree of genetic and behavioural plasticity [51, 52]. Ultimately, field trials are required to determine the impact against natural populations. Future SFS studies can also address whether the repellent works against insecticide resistant mosquito populations [36, 53] and assess whether there is an added protective effect of using a repellent device in combination with other tools deployed in homes such as LLINs and IRS.

Simulations demonstrated that a uniform distribution of metofluthrin concentration is found inside the hut and given the reduction in mosquito activity, the threshold concentration previously determined for *Anopheles quadrimaculatus* mosquitoes appears to be effective for *An. gambiae* mosquitoes. SR concentration is highly affected by air movement, thus outdoor protective concentrations may be found close to the hut or below the eave levels where air flow is minimized. This effect was shown by the minimal impact seen on outdoor host-seeking collections when devices were placed in the eaves.

## Conclusions

This semi-field trial demonstrated that indoor deployment of as few as four novel slow-release spatial repellent emanating devices reduced overall indoor density of anopheline mosquitoes when evaluated overnight. When huts were occupied, however, light trap collections of mosquitoes, used as a proxy for host-seeking rates, were greater with devices present. The reduced indoor density of mosquitoes when CRDs were deployed was attributed to lower collections of indoor resting mosquitoes found alive the next morning. Statistically, this effect was shown, when comparing the effect of occupied (Experiment 3) versus unoccupied huts (Experiment 2) to extract the influence of the human volunteer, while CRDs show increasing efficacy. This effect is likely due to the elicited excito-repellency effects and high concentration of repellent in the small space of the huts used in these experiments. Modelled concentration distribution beyond the threshold concentration for protection was found to be a fair indicator of the effective repellency of these devices. Next studies will investigate the impact of CRDs on mosquito house entry, exit, foraging and feeding to further understand mosquito dynamics with repellents. Additional studies will also focus on the epidemiological impact of CRDs within large cohorts to determine the protective efficacy of CRDs and longevity of protection against malaria vectors.

## Additional file

**Additional file 1.** Multivariable analysis of impact of CRDs on indoor host-seeking of mosquitoes.

## Abbreviations

AI: active ingredient
BG: Biogents
CDC: Centers for Disease Control and Prevention
CFD: computational fluid dynamics
CI: confidence interval
CRD: controlled-release spatial repellent device
GLM: generalized linear model
IRS: indoor residual spraying
ITN: insecticide-treated net
LLIN: long-lasting insecticide-treated nets
MEMS: micro-electro-mechanical-systems
MRT: Macha Research Trust
OR: odds ratio
SFS: semi-field system
SR: spatial repellent
WHO: World Health Organization
