# Supplementary material for: Controlled release spatial repellent devices (CRDs) as novel tools against malaria transmission: a semi-field study in Macha, Zambia
Source: Malar J. 2018 Nov 26;17:437. doi: 10.1186/s12936-018-2558-0 (PMC6258499; doi:10.1186/s12936-018-2558-0)
Supplement: Supplementary file 1 — Additional file 1. Multivariable analysis of impact of CRDs on indoor host-seeking of mosquitoes. [file 12936_2018_2558_MOESM1_ESM.docx]

Additional file 1: Multivariable analysis of impact of CRDs on indoor foraging of mosquitoes.

|  | Odds ratio | 95% CI | p |
| --- | --- | --- | --- |
|  |  |  |  |
| Treatment | 1.31 | 1.12, 1.54 | **0.001** |
|  |  |  |  |
| Experiment^*^ 2  1  3 | Ref  0.06 | 0.03, 0.12 | **0.001** |
|  | 4.88 | 3.59, 6.61 | **0.001** |
| Mean temp | 1.31 | 1.11, 1.55 | **0.001** |

^*^ ^Experiment 2 deployed 4 devices hanging from rafters of hut, huts unoccupied, Experiment 1 deployed 12 CRDs in the eaves and four hanging from rafters, hut unoccupied. Experiment 3 deployed 4 devices hanging from rafters of hut, huts unoccupied.^

^†Mean temp refers to the mean night temperature recorded during the time of the experiment.^

Odds ratios were generated from generalized linear models (GLMs) using a Poisson distribution with logit link function comparing of number of mosquitoes collected from indoor light traps, with or without the CRD, accounting for experiment type, environmental variables and the timing of each experiment. For each experiments, CRDs were alternated in a cross-over design and impacts on released female *An. gambiae s.s.* mosquitoes studied over 10 experimental nights.
